# Chronic kidney disease attenuates the impact of obesity on quality of life

**DOI:** 10.1038/s41598-020-59382-9

**Published:** 2020-02-11

**Authors:** Sang Heon Suh, Hong Sang Choi, Chang Seong Kim, Eun Hui Bae, Seong Kwon Ma, Dae Ho Lee, Soo Wan Kim

**Affiliations:** 1 Department of Internal Medicine, Chonnam National University Medical School, Gwangju, Korea; 2 Department of Internal Medicine, Gachon University College of Medicine, Incheon, Republic of Korea

**Keywords:** End-stage renal disease, Glomerulus

## Abstract

The impact of obesity on health-related quality of life (HRQoL) in chronic kidney disease (CKD) population has not been elucidated, despite the impairment of HRQoL in the obese among general population. We hypothesized that the impact of obesity on HRQoL might be confounded by impaired renal function in CKD population, and that CKD would attenuate the impact of obesity on HRQoL. To compare the impact of obesity on HRQoL according to kidney function, 17,001 subjects from Korea National Health and Nutrition Examination Survey (2008–2011) were categorized by estimated glomerular filtration rate (eGFR), as follows: group 1, eGFR ≥ 90 mL/min/1.73 m^2^; group 2, eGFR of 60–89 mL/min/1.73 m^2^; group 3, eGFR < 60 mL/min/1.73 m^2^. The association between obesity parameters (body mass index, waist circumference and, truncal fat mass) and HRQoL parameters (EQ-5D index and EQ-VAS) were cross-sectionally analyzed. Despite robust correlations between obesity parameters and low EQ-5D index or EQ-VAS in general population, no significant association was observed in group 3 population. Impact of obesity on HRQoL in CKD population was only limitedly observed in the mobility domain of EQ-5D, as mobility limitation was associated with increased body mass index or waist circumference regardless of kidney function. Therefore, the impact of obesity on HRQoL seems significantly attenuated in CKD population, suggesting that the risk of obesity should not be over-estimated in patients with CKD, especially with respect to HRQoL.

## Introduction

Obesity imposes a great burden on the public health globally, as it increases the risk of type 2 diabetes mellitus, cardiovascular disease, cerebrovascular accident, and malignancy, and is subsequently associated with high cardiovascular and all-cause mortalities^1–3^. Especially, central obesity, defined by waist circumference (WC) or WC to height ratio, more precisely predicts cardiovascular risk factors than body mass index (BMI) does^4^. The concept, metabolically healthy obesity, introduced broad spectral phenotypes of obesity and emphasized the importance of anatomical distribution of body fat^5^. Indeed, various indices from direct measurement of regional fat mass^6^ or from calculation of more than 2 anatomical fat deposits^7^ have proved their superiority over BMI on estimating the risk of cardiovascular events. On the basis of conceptual advances, novel links between obesity and human diseases are now being uncovered.

Kidney is a target of obesity^8^, as evidences suggest that conditions related to obesity, such as insulin resistance^9^, up-regulation of leptin^10^, or down-regulation of adiponectin^11^, promote glomerular injury and albuminuria, leading to the decline of kidney function. Nevertheless, clinical studies have not concluded the impact of obesity on CKD progression. A cohort study including more than 450,000 nondialysis-dependent subjects reported U-shaped association of BMI and clinical outcomes with the best in overweight and mildly obese patients, rather than in patients with ideal body weight^12^. Another cohort study including 1,940 participants with CKD presented quite conflicting results that obesity (defined as BMI ≥ 25 kg/m^2^) and/or concurrent metabolic abnormalities accelerate CKD progression^13^. These inconsistent results strongly imply the complex nature of CKD, where findings drawn from the general population should not be easily extrapolated.

Health-related quality of life (HRQoL) has emerged as an indispensable clinical outcome^14,15^. Despite the vague definition and essentially subjective nature of HRQoL^16^, several questionnaires have been developed to quantify HRQoL and have been validated in various clinical settings^17,18^. Previous studies reported relatively consistent results that obesity impairs HRQoL^19–21^, while the association of CKD with poor HRQoL has been an issue of debate^22–25^, until a recent study including more than 46,000 participants reported a significant association between CKD and poor HRQoL^26^, suggesting that the impact of obesity on HRQoL might be confounded by impaired renal function in CKD population. Yet, the association of obesity and HRQoL in subjects with kidney dysfunction has never been studied. We, therefore, hypothesized that CKD would attenuate the impact of obesity on HRQoL. In the present study, taking advantage of a nationwide, community-based population study, we analyzed and compared the impact of obesity on HRQoL among the subgroups stratified by eGFR.

## Results

### Baseline characteristics

**Figure 1:**
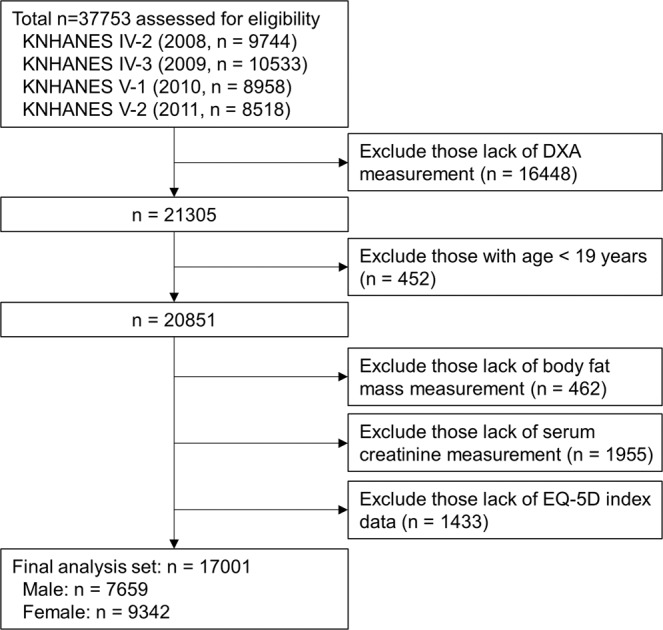
Flow chart of study participants. Abbreviation: DXA, dual-energy X-ray absorptiometry; eGFR, estimated glomerular filtration rate; KNHANES, Korea National Health and Nutrition Examination Survey.

To compare the baseline characteristics according to the kidney function, all study subjects were divided into 3 groups (Fig. 1 and Table 1). Among the entire study participants, 2.74% belonged to group 3, while 62.12% and 32.14% did to group 1 and group 2, respectively. The study subjects were older in age as kidney function deteriorated. The surrogates of HRQoL, EuroQoL-5 dimension-3 level (EQ-5D) index and EuroQoL-visual analogue scale (EQ-VAS) scores, significantly decreased as kidney dysfunction progressed. Compared to group 1, the proportion of subjects with mobility limitation sharply increased in in groups 2 and 3 (10.42% vs. 26.34% and 49.68%, respectively). Obesity parameters also showed robust distinctions according to the renal function. Compared to the subjects at groups 1, BMI and other indices regarding central obesity such as waist circumference (WC), truncal fat mass (TF) as well as ratio of TF to whole body fat mass (TF/WF) increased in the subjects of groups 2 and 3. The ratio of leg fat mass to WF (LF/WF) decreased as kidney function deteriorated, indicating the predominance of central obesity in CKD populations. The subjects of groups 2 and 3 also had a higher prevalence of comorbidities, and showed remarkable distinction in laboratory findings. Compared to the other two groups, hemoglobin level decreased, while serum ferritin level increased in group 3. Metabolic derangement became apparent as eGFR declined, as serum triglyceride, fasting glucose, alkaline phosphatase, and parathyroid hormone levels increased in groups 2 and 3, compared to group 1. The incidence of proteinuria also increased as kidney function deteriorated.

**Table 1:** Baseline characteristics of the study population categorized by eGFR.

(mL/min./1.73 m^2^)	Group 1 (eGFR ≥ 90)	Group 2 (60 ≤ eGFR < 90)	Group 3 (eGFR < 60)	*p* value
**Demographics**				
Sample numbers (%)	11071 (62.120)	5465 (32.145)	465 (2.735)	
Male sex	4565 (41.234)	2849 (52.132)	246 (52.903)	<0.001
Age	43.718 ± 13.376	60.790 ± 13.146	70.794 ± 8.446	<0.001
**HRQoL indices**				
EQ-5D index	0.953 ± 0.097	0.907 ± 0.151	0.834 ± 0.199	<0.001
EQ-VAS score	75.164 ± 16.133	71.905 ± 19.792	67.672 ± 20.795	<0.001
Mobility limitation	1154 (10.424)	1440 (26.349)	231 (49.677)	<0.001
**Obesity parameters**				
BMI (kg/m^2^)	23.467 (3.361)	24.061 (3.125)	24.499 (3.289)	<0.001
WC (cm)	80.153 (9.858)	83.714 (9.341)	86.557 (9.458)	<0.001
Truncal fat (g)	9000.809 (3551.934)	9470.456 (3432.993)	10350.732 (3416.555)	<0.001
TF/WF	0.507 (0.068)	0.542 (0.058)	0.564 (0.055)	<0.001
LF/WF	0.320 (0.061)	0.287 (0.049)	0.269 (0.045)	<0.001
**Co-morbidities**				
Smoking	4167 (37.683)	2496 (45.790)	242 (52.043)	<0.001
Diabetes	568 (5.131)	676 (12.370)	150 (32.258)	<0.001
Hypertension	1553 (14.028)	1944 (35.572)	331 (71.183)	<0.001
Dyslipidemia	860 (7.768)	719 (13.156)	96 (20.645)	<0.001
CAD	115 (1.039)	202 (3.696)	52 (11.183)	<0.001
Stroke	96 (0.867)	193 (3.532)	50 (10.753)	<0.001
COPD	44 (0.490)	45 (0.863)	6 (1.345)	0.0086
Liver cirrhosis	19 (0.172)	17 (0.311)	1 (0.215)	0.1651
Depression	1562 (14.109)	822 (15.044)	74 (15.914)	0.1823
**Laboratory findings**				
eGFR (mL/min./1.73 m^2^)	104.869 ± 9.981	80.042 ± 7.668	48.871 ± 10.809	<0.001
Hemoglobin (g/dL)	13.917 ± 1.582	14.115 ± 1.518	13.138 ± 1.869	<0.001
Ferritin (ng/mL)	81.572 ± 113.916	100.572 ± 139.713	114.478 ± 127.443	<0.001
Triglyceride (mg/dL)	130.187 ± 114.960	146.125 ± 104.011	169.869 ± 107.895	<0.001
Glucose (mg/dL)	95.949 ± 21.442	101.486 ± 24.332	109.725 ± 36.306	<0.001
Alkaline phosphatase (IU/L)	218.818 ± 69.663	239.562 ± 74.111	249.923 ± 82.133	<0.001
Parathyroid hormone (pg/mL)	65.226 ± 28.570	66.938 ± 26.581	85.341 ± 54.289	<0.001
Urine protein ≥ 1+	765 (6.910)	410 (7.689)	103 (23.146)	<0.001

Note: Values for categorical variables are given as number (percentage); values for continuous variables, as mean ± standard deviation. eGFR was calculated using the CKD-EPI creatinine equation. Abbreviations: BMI, body mass index; CAD, coronary artery disease; COPD, chronic obstructive pulmonary disease; eGFR, estimated glomerular filtration rate; LF/WF, ratio of leg fat mass to whole body fat mass; TF/WF, ratio of truncal fat mass to whole body fat mass; WC, waist circumference.

### Modification of association between obesity and HRQoL by kidney function

To investigate the association between obesity and HRQoL, survey-weighted generalized linear model was analyzed (Table 2). In the analyses of crude and adjusted models 1–3 including all study subjects, BMI was negatively associated with EQ-5D index score, which trend was substantially attenuated as estimated glomerular filtration rate (eGFR) decreased. In the subgroup analysis of group 1 subjects, the similar negative association was still observed, even after full adjustments with co-variates (Coefficients, −0.002; 95% CI, −0.004–-0.001; P = 0.0033). This association was marginally significant in group 2 subjects (Coefficients, −0.002; 95% CI, −0.004–0; P = 0.0185), but was no more valid in group 3 subjects (Coefficients, −0.005; 95% CI, −0.011–0.001; P = 0.0985). Further, neither of WC, TF, nor TF/WF was significantly associated with EQ-5D index among group 3 population either in crude or adjusted models, although the statistical significance was observed in the analyses including all study population. To figure out whether differential anatomical fat deposit other than central obesity may be linked to HRQoL in CKD, the effect of LF/WF on EQ-5D index was also analyzed. Despite a significant association between LF/WF and EQ-5D index was robustly present even after full adjustment of co-variates in the analysis of all study subjects and in subgroup analyses of group 1 and 2 populations., the association of LF/WF with EQ-5D index no longer existed in the analysis of adjusted model 3 for group 3 population. As the number of subjects in group 3 were substantially smaller than those of the others, the study subjects were dichotomized by incidence of CKD for propensity score-matching analysis (Table S1), where none of BMI, WC or TF was significantly associated with EQ-5D, regardless of incident CKD (Table S2). EQ-VAS score in the analysis of fully adjusted model including group 3 population was not associated with either of BMI, WC, or TF (Table S3). Taken together, these indicate that the association between obesity and HRQoL is modified by kidney function.

**Table 2:** Mean differences of the EQ-5D index according to obesity parameters in all study subjects and in subgroups stratified by eGFR categories.

		Crude		Adjusted for model 1		Adjusted for model 2		Adjusted for model 3	
		Coefficients (95%CIs)	*P* value	Coefficients (95%CIs)	*P* value	Coefficients (95%CIs)	*P* value	Coefficients (95%CIs)	*P* value
BMI	All subjects	−0.002 (−0.002, −0.001)	<0.001	−0.001 (−0.002, 0)	<0.001	−0.001 (−0.001,0)	0.0659	−0.002 (−0.003, −0.001)	<0.001
	Group 1	−0.002 (−0.002, −0.001)	<0.001	−0.001 (−0.002, 0)	0.0015	−0.001 (−0.001, 0)	0.0156	−0.002 (−0.004, −0.001)	0.0033
	Group 2	0 (−0.001, 0.002)	0.7874	−0.001 (−0.003, 0)	0.0234	−0.001 (−0.002, 0.001)	0.2254	−0.002 (−0.004, 0)	0.0185
	Group 3	−0.003 (−0.008, 0.001)	0.1675	−0.004 (−0.009, 0.001)	0.0874	−0.004 (−0.009, 0.002)	0.2288	−0.005 (−0.011, 0.001)	0.0985
WC	All subjects	−0.001 (−0.001, −0.001)	<0.001	−0.001 (−0.001, 0)	<0.001	0 (−0.001, 0)	<0.001	−0.001 (−0.002, −0.001)	<0.001
	Group 1	−0.001 (−0.001, −0.001)	<0.001	−0.001 (−0.001, 0)	<0.001	−0.001 (−0.001, 0)	<0.001	−0.001 (−0.002, −0.001)	<0.001
	Group 2	0 (−0.001, 0)	0.0916	−0.001 (−0.001, 0)	0.0034	0 (−0.001, 0)	0.0834	−0.001 (−0.002, 0)	0.0085
	Group 3	−0.001 (−0.002, 0.001)	0.5619	−0.002 (−0.004, 0)	0.0507	−0.001 (−0.004, 0.001)	0.1888	−0.002 (−0.004, 0.001)	0.1472
TF	All subjects	−0.003 (−0.003, −0.002)	<0.001	−0.001 (−0.001,0)	0.0229	0 (−0.001, 0.001)	0.8216	−0.001 (−0.002, 0)	0.2608
	Group 1	−0.002 (−0.003, −0.002)	<0.001	−0.001 (−0.001, 0)	0.0023	0 (−0.001, 0)	0.119	−0.002 (−0.003, 0)	0.0262
	Group 2	−0.002 (−0.004, −0.001)	0.0026	0 (−0.001, 0.001)	0.8465	0.001 (−0.001, 0.002)	0.3186	0 (−0.002, 0.002)	0.8347
	Group 3	−0.006 (−0.012, −0.001)	0.0161	−0.004 (−0.009, 0.001)	0.108	−0.002 (−0.008, 0.003)	0.4196	−0.004 (−0.01, 0.001)	0.1224
TF/WF	All subjects	−0.134 (−0.158, −0.109)	<0.001	0.04 (0.012, 0.069)	0.0057	0.083 (0.05, 0.116)	<0.001	0.127 (0.056, 0.197)	<0.001
	Group 1	−0.088 (−0.113, −0.064)	<0.001	0 (−0.03, 0.031)	0.9852	0.027 (−0.008, 0.061)	0.1292	0.075 (−0.008, 0.175)	0.0761
	Group 2	0.025 (−0.047, 0.097)	0.4964	0.085 (0.012, 0.158)	0.0228	0.142 (0.063, 0.221)	<0.001	0.151 (0.045, 0.257)	0.0055
	Group 3	0.335 (0.025, 0.645)	0.0349	0.154 (−0.172, 0.481)	0.3553	0.32 (−0.02, 0.66)	0.0663	0.138 (−0.215, 0.49)	0.446
LF/WF	All subjects	0.14 (0.113, 0.168)	<0.001	−0.052 (−0.089, −0.014)	0.008	−0.101 (−0.141, −0.061)	<0.001	−0.163 (−0.249, −0.078)	<0.001
	Group 1	0.09 (0.062, 0.118)	<0.001	0.002 (−0.04, 0.045)	0.9248	−0.031 (−0.074, 0.011)	0.1521	−0.107 (−0.207, −0.007)	0.0368
	Group 2	−0.06 (−0.143, 0.023)	0.1591	−0.116 (−0.21, −0.022)	0.0157	−0.188 (−0.288, −0.089)	<0.001	−0.19 (−0.322, −0.058)	0.0049
	Group 3	−0.637 (−1.066, −0.208)	0.0039	−0.334 (−0.774, 0.107)	0.139	−0.549 (−1.027, −0.072)	0.0251	−0.345 (−0.876, 0.186)	0.2039

Note: Model 1, adjusted for age and sex. Model 2, Model 1 + adjusted for comorbidities (diabetes, hypertension, dyslipidemia, coronary artery disease, stroke, chronic obstructive pulmonary disease, and smoking). Model 3, model 2 + adjusted for laboratory finding (hemoglobin, ferritin, fasting plasma glucose and triglyceride, parathyroid hormone, alkaline phosphatase, proteinuria, and eGFR). Abbreviations: BMI, body mass index; CI, confidence interval; Group 1, ≥90 mL/min/1.73 m^2^; Group 2, eGFR of 60–89 mL/min/1.73 m^2^; Group 3, eGFR < 60 mL/min/1.73 m^2^; LF/WF, ratio of leg fat mass to whole body fat mass; TF/WF, ratio of truncal fat mass to whole body fat mass; WC, waist circumference.

To test the impact of obesity parameters on HRQoL in the elderly and in the relative young, we additionally analyzed the effect of BMI, WC or TF on EQ-VAS score in subgroups stratified by age categories (Table S4), as the subgroup with impaired renal function was significantly older in the ages (Table 1). Only BMI in the subgroup with age <60 proved a statistically significant association with EQ-VAS score in the fully adjusted model, albeit the trend was inversed from the crude analysis model, suggesting the beneficial effect of high BMI in the subjects with chronic illness.

### Disappearance of inverted U-shaped correlation between BMI and HRQoL in CKD stages 3–5 population

**Figure 2:**
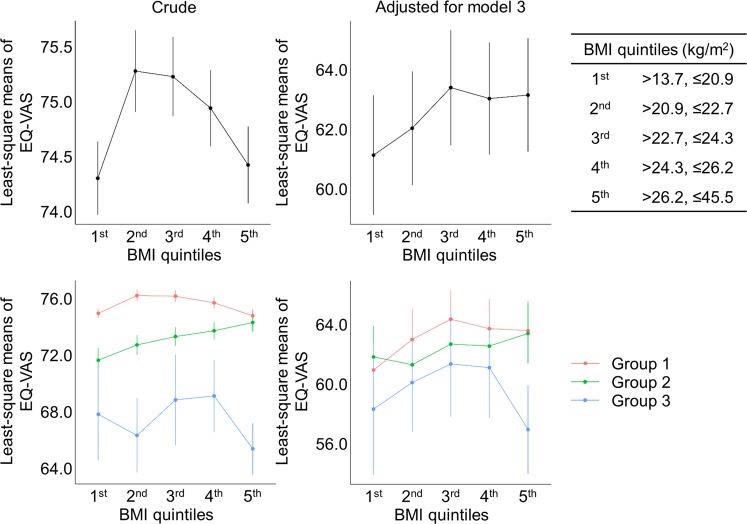
Comparison of least-square means of EQ-5D index among BMI quintiles in all study subjects and in subgroups stratified by eGFR categories. Abbreviations: BMI, body mass index; Group 1, ≥90 mL/min/1.73 m^2^; Group 2, eGFR of 60–89 mL/min/1.73 m^2^; Group 3, eGFR <60 mL/min/1.73 m^2^.

**Figure 3:**
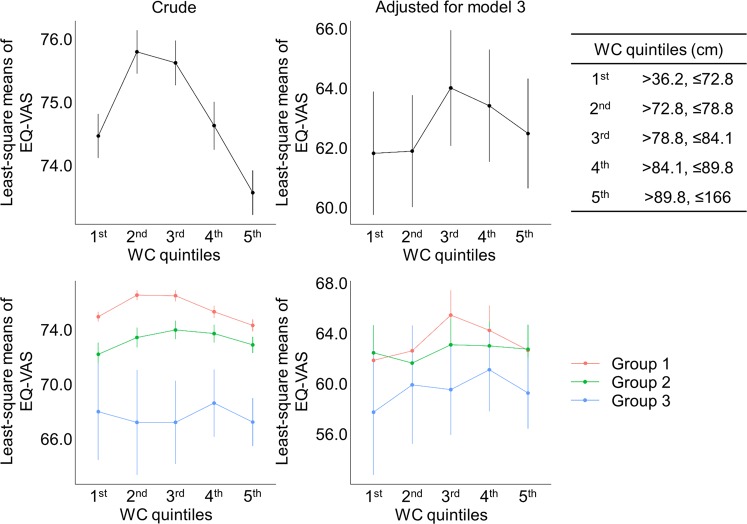
Comparison of least-square means of EQ-5D index among WC quintiles in all study subjects and in subgroups stratified by eGFR categories. Abbreviations: WC, waist circumference; Group 1, ≥90 mL/min/1.73 m^2^; Group 2, eGFR of 60–89 mL/min/1.73 m^2^; Group 3, eGFR <60 mL/min/1.73 m^2^; WC, waist circumference.

**Figure 4:**
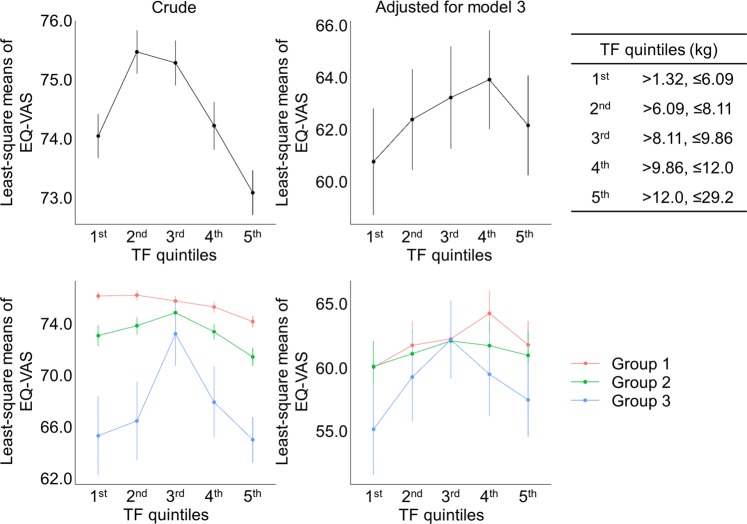
Comparison of least-square means of EQ-5D index among TF quintiles in all study subjects and in subgroups stratified by eGFR categories. Abbreviations: TF, truncal fat mass; Group 1, ≥90 mL/min/1.73 m^2^; Group 2, eGFR of 60–89 mL/min/1.73 m^2^; Group 3, eGFR < 60 mL/min/1.73 m^2^; TF, truncal fat mass.

To examine whether a non-linear correlation between BMI and HRQoL score may be present, such as U-shaped or inverted J-shaped correlation, the study subjects were divided into quintiles according to BMI (Fig. 2, Tables 3, and S5). In the crude analysis including all subjects, inverted U-shaped correlation was clearly observed between BMI and EQ-VAS score, where the score was lowest in the 1st quintile BMI group, peaked in the 2nd and 3rd quintile BMI groups, and then dropped in 4th and 5th quintile BMI groups. In the analysis of fully adjusted model including all subjects, however, the inverted U-shaped correlation no more existed, where the score was lowest in the 1st quintile BMI group, peaked in 3rd quintile BMI groups, and plateaued in in 4th and 5th quintile BMI groups. The similar pattern was observed in the subgroup analyses of group 1 subjects. Intriguingly, in the subgroup analyses of crude model including group 2 subjects, EQ-VAS score continued to rise as BMI increased with its peak in the 5th quintile BMI group, although the statistical significance disappeared after adjustment of covariates. Moreover, no significant differences of EQ-VAS scores along the entire BMI ranges either in the crude or adjusted analysis including group 3 population. To additionally address the probable non-linear correlation of HRQoL with other obesity-related parameters, the study subjects were divided into quintiles according to WC or TF. The baseline characteristics of the study population stratified by WC and TF quintile categories are shown in Tables S6 and S7, respectively. In the analyses of crude and adjusted model including all study subjects or group 1 subjects, inverted U-shaped correlation was observed between WC and EQ-VAS score (Fig. 3 and Table S8). In the analyses including group 2 and 3 populations, however, no remarkable differences in the EQ-VAS score were observed along the entire range of WC quintiles. On the other hand, inverted J-shaped correlation was observed between TF and EQ-VAS score in the analysis of crude model including all study subjects or group 1 subjects, with a sharp decline of EQ-VAS score in the 4th and/or 5th TF quintiles (Fig. 4 and Table S9). Of note, after full adjustment of covariates, such a sharp decline of EQ-VAS score was markedly blunted with a peak of EQ-VAS score in the 4th quintile. Again, no distinct alterations in the EQ-VAS score were observed along the entire range of TF quintiles from the analysis of adjusted model including group 3 subjects. Therefore, these collectively suggest that, despite a unique association between central obesity and HRQoL in general populations, even a non-linear correlation does not exist in the subjects with impaired kidney function.

**Table 3:** Mean differences of the EQ-VAS score according to BMI quintiles in all study subjects and in subgroups stratified by eGFR categories.

	Crude		Adjusted for model 3	
	Coefficients (95%CIs)	*p* value	Coefficients (95%CIs)	*p* value
**All subjects**				
BMI, 1st quintile	Reference		Reference	
BMI, 2nd quintile	0.963 (0.085, 1.84)	0.0319	1.128 (−0.609, 2.866)	0.2037
BMI, 3rd quintile	0.913 (0.026, 1.799)	0.0441	2.543 (0.87, 4.217)	0.003
BMI, 4th quintile	0.629 (−0.282, 1.541)	0.1764	2.234 (0.501, 3.967)	0.0118
BMI, 5th quintile	0.119 (−0.8, 1.039)	0.7996	2.341 (0.458, 4.225)	0.0152
**Group 1**				
BMI, 1st quintile	Reference		Reference	
BMI, 2nd quintile	1.25 (0.268, 2.232)	0.0129	1.912 (−0.582, 4.406)	0.1335
BMI, 3rd quintile	1.205 (0.219, 2.191)	0.017	3.33 (1.047, 5.614)	0.0044
BMI, 4th quintile	0.738 (−0.278, 1.754)	0.1552	2.773 (0.343, 5.204)	0.0258
BMI, 5th quintile	−0.167 (−1.219, 0.886)	0.7561	2.379 (−0.055, 4.812)	0.056
**Group 2**				
BMI, 1st quintile	Reference		Reference	
BMI, 2nd quintile	1.08 (−0.944, 3.105)	0.2961	0.018 (−2.523, 2.559)	0.989
BMI, 3rd quintile	1.666 (−0.432, 3.765)	0.1202	1.58 (−1.005, 4.165)	0.2314
BMI, 4th quintile	2.071 (0.043, 4.1)	0.0459	1.468 (−1.046, 3.982)	0.253
BMI, 5th quintile	2.644 (0.54, 4.747)	0.0141	2.381 (−0.38, 5.142)	0.0916
**Group 3**				
BMI, 1st quintile	Reference		Reference	
BMI, 2nd quintile	−1.487 (−9.094, 6.119)	0.7019	0.604 (−7.784, 8.991)	0.888
BMI, 3rd quintile	0.997 (−7.876, 9.87)	0.8259	1.729 (−7.421, 10.879)	0.7115
BMI, 4th quintile	1.279 (−6.509, 9.066)	0.7479	0.531 (−9.026, 10.089)	0.9134
BMI, 5th quintile	−2.425 (−9.638, 4.788)	0.5105	−3.597 (−12.216, 5.022)	0.4144

Note: Model 3, adjusted for age, sex (Model 1), comorbidities (diabetes, hypertension, dyslipidemia, coronary artery disease, stroke, chronic obstructive pulmonary disease, and smoking, Model 2), and laboratory finding (hemoglobin, ferritin, fasting plasma glucose and triglyceride, parathyroid hormone, alkaline phosphatase, proteinuria, and eGFR). Abbreviations: BMI, body mass index; CI, confidence interval; Group 1, ≥90 mL/min/1.73 m^2^; Group 2, eGFR of 60–89 mL/min/1.73 m^2^; Group 3, eGFR < 60 mL/min/1.73 m^2^.

### Impact of obesity on mobility limitation in subjects with renal dysfunction

To unveil the association between obesity and mobility, a component of HRQoL, which has been suggested in certain populations^27,28^, odd ratios (ORs) of obesity-related parameters for mobility limitation were analyzed (Table 4). In the analyses of crude and adjusted models including all study subjects or group 1 and 2 subjects, the association of mobility limitation with BMI or WC was significantly observed. Interestingly, the significant correlation still existed between BMI (OR, 1.152; 95% CI, 1.054–1.259; P = 0.0021) or WC (OR, 1.05; 95% CI, 1.02–1.081; P = 0.0013) and mobility limitation even in the analysis of adjusted model including group 3 subjects, although the statistical significances were not observed in the analyses of crude models. The association between increased TF and mobility limitation was significantly observed only in the analyses of adjusted models for group 1 and group 3 subjects, despite the significant associations in the crude analyses of all study participants and all subgroups according to eGFR. These results suggest that the impact of obesity on HRQoL is only limited to mobility among subjects with renal dysfunction.

**Table 4:** Odd ratio of mobility limitation according to body composition parameters in all study subjects and in subgroups stratified by eGFR categories.

	Crude		Adjusted for model 3	
	OR (95%CIs)	*p* value	OR (95%CIs)	*p* value
**BMI**				
All subjects	1.06 (1.038, 1.082)	<0.001	1.08 (1.055, 1.104)	<0.001
Group 1	1.083 (1.047, 1.119)	<0.001	1.085 (1.046, 1.125)	<0.001
Group 2	1.034 (1.004, 1.065)	0.0276	1.073 (1.041, 1.106)	<0.001
Group 3	1.071 (0.998, 1.15)	0.0595	1.152 (1.054, 1.259)	0.0021
**WC**				
All subjects	1.022 (1.015, 1.029)	<0.001	1.03 (1.022, 1.038)	<0.001
Group 1	1.034 (1.021, 1.046)	<0.001	1.039 (1.025, 1.054)	<0.001
Group 2	1.008 (0.999, 1.018)	0.0811	1.021 (1.011, 1.032)	<0.001
Group 3	1.017 (0.993, 1.041)	0.1746	1.05 (1.02, 1.081)	0.0013
**TF**				
All subjects	1.069 (1.05, 1.089)	<0.001	1.041 (1.02, 1.064)	<0.001
Group 1	1.086 (1.053, 1.12)	<0.001	1.063 (1.026, 1.102)	<0.001
Group 2	1.049 (1.021, 1.077)	<0.001	1.02 (0.989, 1.052)	0.2143
Group 3	1.098 (1.023, 1.177)	0.01	1.126 (1.035, 1.226)	0.0064

Note: Model 3, adjusted for age, sex (Model 1), comorbidities (diabetes, hypertension, dyslipidemia, coronary artery disease, stroke, chronic obstructive pulmonary disease, and smoking, Model 2), and laboratory finding (hemoglobin, ferritin, fasting plasma glucose and triglyceride, parathyroid hormone, alkaline phosphatase, proteinuria, and eGFR). Abbreviations: BMI, body mass index; CI, confidence interval; Group 1, ≥90 mL/min/1.73 m^2^; Group 2, eGFR of 60–89 mL/min/1.73 m^2^; Group 3, eGFR < 60 mL/min/1.73 m^2^; OR, odd ratio; TF, truncal fat; WC, waist circumference.

## Discussion

In the present study, we analyzed the association of obesity and HRQoL in general population and in subgroups categorized by kidney function. Contrary to the findings in general population, the impact of obesity on HRQoL was markedly attenuated as kidney function declined. Even a non-linear, inverted U-shaped association between obesity and HRQoL was not valid in subjects with kidney dysfunction. The association of obesity with HRQoL in CKD population was only limited to the mobility dimension, a component of EQ-5D, where obesity significantly impaired mobility both in general and CKD populations.

Considering the contribution of obesity to CKD progression^13^, loss of correlation between obesity and HRQoL in CKD population was unexpected. Of interest is that a similar trend has been reported from the studies of serum uric acid level in CKD population^29,30^. Based on the studies devoid of specific subgroup analyses for CKD population^31,32^, it has been widely accepted that hyperuricemia is a risk factor for cardiovascular and all-cause mortality. Although hyperuricemia accelerates CKD progression^33^, higher uric acid levels were associated with lower risk of all-cause and cardiovascular mortality in the hemodialysis population^29^. This paradoxical association is an example of reverse epidemiology, which is well-described among patients on hemodialysis in respect of hypertension^34^, total cholesterol level^35^, plasma total homocysteine level^36^, and leptin^37^. While the precise mechanism has not been established, statistical analyses are indicating the role of systemic inflammation and/or malnutrition as a cause of these paradoxical associations^29,35–37^. For example, increment of total cholesterol level was associated with a decreased risk of all-cause mortality in all HD patients and in HD patients with inflammation/malnutrition, whereas serum cholesterol level was associated with an increased risk in the absence of inflammation/malnutrition^35^. Accordingly, given that low BMI and total body fat percentage are components of suggested criteria for malnutrition in CKD patients, and that obesity does not necessarily mean well-nourished status^38^, it is speculated that the mild obesity in CKD population might be a proof of adequate nutrition without active inflammation, and might substantially abolish the drawbacks of obesity observed in subjects with normal kidney function, leading to preserved HRQoL outcomes. Another explanation for loss of correlation between obesity and HRQoL in CKD population is the possible role of metabolic abnormalities. A recent study^39^ reported the association of obesity phenotypes and HRQoL in patients with CKD stages 3–4, which revealed that HRQoL was impaired in the metabolically unhealthy obese, but not in the metabolically healthy obese, patients. The results clearly suggest that obesity defined by anthropometric measurement has its limitation in the predictability of HRQoL in CKD patients, and emphasize the impact of metabolic status based on the laboratory findings. To summarize, the findings from the current study and others suggest that obesity *per se* might not impair HRQoL in CKD population, and that, rather, inflammation or metabolic abnormalities would be truly associated with low HRQoL, both of which conditions are prevalent and easily accompanied in patients with CKD^13,38^.

Various parameters other than BMI, such as WC, TF, TF/WF, and LF/WF, were comprehensively analyzed to reveal the association of obesity or anatomical fat deposit status with HRQoL in general population and in subgroups with kidney dysfunction, as distinct features of each index might be reported regarding HRQoL^7^. While the overall trend was similar regarding the correlation with HRQoL, the individual parameters also had distinct features in detail (Figs. 2–4). The shape of correlation curves between HRQoL and each of BMI, WC, and TF in adjusted models was unique among group 3 population, with inverted U-shapes in BMI and TF, and relatively flat shape in WC. Although none of them were statistically significant, these results imply that a certain index related to obesity or anatomical fat deposit, but not analyzed in this study, might be superior to the prediction of HRQoL and the guidance of nutritional support in CKD population, which should be further highlighted in the future.

Contrary to the overall EQ-5D index, the mobility limitation, a dimension of EQ-5D questionnaire, was significantly associated with obesity in group 3 population as well as in general population. A previous study reported the associations of body size and composition with functional ability and HRQoL in patients undergoing HD^40^, where no statistical significance was observed between HRQoL index and adiposity measures, such as BMI, WC, and intra-abdominal fat. These conflicting findings may result from the different study population, as the previous study included subjects with end-stage renal diseases only, while the subjects in the current study are not restricted to dialysis-dependents individuals. Considering the trend of association between overall EQ-5D index and obesity according to CKD stages, it seems quite reasonable that impact of obesity on the mobility also attenuates as CKD progresses form earlier stages to end stage. Hence, our results collectively suggest that medical interventions targeting obesity control should be recommended in a subset of CKD patients who have physically active lifestyle for better HRQoL outcomes.

There are some limitations to our study. First, we cannot elucidate causal relationships, as a cross-sectional survey data was analyzed. Second, eGFR value may not accurately reflect the actual status of kidney function, since serum creatinine and other laboratory values for each participants were measured only once. Third, no data is available regarding renal replacement therapy in this survey. Therefore, CKD population in the present study may include both nondialysis-dependent and dialysis-dependent subjects. Fourth, the number of study participants in each subgroup is considerably disproportional, although the numbers represent all of non-institutionalized general population in South Korea. Finally, because the cohort of this survey consists of only Koreans dwelling in South Korea, caution is required if our results are to be generalized to other ethnic groups or other nations.

In conclusion, we demonstrated that, compared to that in general population, the association of obesity with HRQoL was significantly modified by kidney function, suggesting that CKD attenuates the impact of obesity on HRQoL. The results in the present study indicate that the risk of obesity should not be over-estimated in patients with CKD, especially with respect to HRQoL.

## Methods

### Study design and participants

The Korea National Health and Nutrition Examination Survey (KNHANES) is a nationwide population-based cross-sectional study of the health and nutritional status of the noninstitutionalized Korean population. It consists of a health questionnaire, physical/laboratory examinations, and nutrition survey. The present study analyzed data obtained from KNHANES 2008 to 2011 (Fig. 1), because the measurement of anatomical fat distribution using dual-energy X-ray absorptiometry (DXA) is included in data from that period. The raw data of KNHANES is open to the registered investigators online (https://knhanes.cdc.go.kr). Written informed consent was obtained from each participant in KNHANES at the time of enrollment. Of 37,753 participants in the health questionnaire and physical/laboratory examination of KNHANES 2008 to 2011, participants lack of DXA measurement (n = 16,448), younger than 20 years (n = 452) were excluded. Participants lack of body fat mass measurement (n = 462) or lack of EQ-5D index (n = 1,433) were also excluded. After excluding participants whose kidney function is not accurately estimated (n = 1,955), finally 17,001 participants were included in the analyses. This study protocol was approved by the Institutional Review Board of Chonnam National University Hospital (CNUH-EXP-2019-205), and was conducted in accordance with the Declaration of Helsinki and its later amendments or comparable ethical standards.

### Assessment of HRQoL

HRQoL was assessed using the Korean version of the EQ-5D health questionnaire modified from its original version in English^18^ and the EQ-VAS, which are included in KNHANES. The EQ-5D descriptive system comprises 5 dimensions (mobility, self-care, usual activities, pain/discomfort, and anxiety/depression), each of which are answered with 3 levels: no problem, some problem, and extreme problem (Fig. S1). The possible combination results in 243 (=3^5^) unique health states. Using a scoring algorithm, EQ-5D index scores are calculated from the combination of answers to the questionnaire, ranging from 1 (optimal health) to −0.171 (worst health state or death)^41^. The EQ-5D index has been widely used to quantify HRQoL both in both general^18^ and specific disease populations, such as subjects with CKD populations^42^. Its validity and reliability have been also demonstrated in Koreans^26^. In this study, subjects with mobility limitation was defined as those who answered with “some problem” or “extreme problem” in mobility dimension of EQ-5D health questionnaire. In EQ-VAS, study participants marked their subjective health status using a visual scale ranging from 0 (the worst imaginable health state) to 100 (the best imaginable health state).

### Measurement of regional fat distribution using DXA

DXA is a gold standard method for the measurement of regional fat distribution^43^. Fat mass and muscle mass were measured using DXA (QDR 4800 A; Hologic Inc., Bedford, MA, USA). TF, LF, and WF as well as the ratio of TF/WF and LF/WF, were analyzed in the present study to define regional fat distribution.

### Anthropometric and clinical measurement

Trained staff members measured the height and weight of study participants. BMI was calculated as weight divided by the height squared. Comorbidities with physician diagnosis were analyzed. Blood samples were collected after at least an 8-hour fast, properly processed, immediately refrigerated, and transported in cold storage to the central laboratory (Neodin Medical Institute, Seoul, Korea) within 24 hours. Proteinuria was defined as albuminuria (≥1+) determined by dipstick urine test.

### Assessment of kidney function

eGFR was calculated from serum creatinine level using the CKD-EPI (CKD Epidemiology Collaboration) equation^44^. The CKD-EPI equation more accurately categorizes risk for death than the MDRD (Modification of Diet in Renal Disease) Study equation^45^ and has also demonstrated its accuracy in Asian populations^46^, including Koreans^47^. All study subjects were classified according to eGFR, as follows: Group 1, ≥90 mL/min/1.73 m^2^; Group 2, eGFR of 60–89 mL/min/1.73 m^2^; Group 3, eGFR <60 mL/min/1.73 m^2^. CKD was defined as the presence of proteinuria or eGFR <60 mL/min/1.73 m^2^.

### Statistical analysis

Data are presented as the mean ± standard deviation for continuous variables, and as number, or percent for categorical variables. To compare the difference in the baseline characteristics according to kidney function, one-way analysis of variance and χ2 test were used for continuous and categorical variables, respectively. The composite sample weight was introduced in the analyses to estimate the noninstitutionalized Korean population (Table S10). Survey weight for the participants in the health questionnaire and physical/laboratory examinations was computed using the sampling rate, response rate, and age/sex proportion of the Korean population. Differences in HRQoL according to various indices related to obesity and regional fat distribution were evaluated in models with different variables in all or subsets of study participants. Starting with a crude model, blocks of variables were sequentially introduced to evaluate their effects on the association between HRQoL and obesity. Model 1 was adjusted for age and sex. Model 2 was additionally adjusted for comorbidities (diabetes, hypertension, dyslipidemia, coronary artery disease, stroke, chronic obstructive pulmonary disease, and smoking). Model 3 was further adjusted for laboratory finding (eGFR, hemoglobin, ferritin, fasting plasma glucose and triglyceride, 25-hydroxyvitamin D, parathyroid hormone, alkaline phosphatase, and proteinuria). Concerned with the co-linearity between the independent variables, hemoglobin and ferritin, we revealed only a weak correlation, with a correlation coefficient of 0.29 (Fig. S2). To minimize bias resulted from the relatively small number of subjects with renal dysfunction, the subjects were dichotomized by incidence of CKD, and were propensity score-matched at a 1:1 ratio, where propensity scores were based on age and sex. Statistical analyses were performed using R software version 3.6.0, with a survey package (https://cran.r-project.org/package=survey) for analyses of complex survey data. P < 0.05 was considered statistically significant.

## Supplementary information

Supplementary information.
