# Supplementary material for: Chronic kidney disease attenuates the impact of obesity on quality of life
Source: Sci Rep. 2020 Feb 11;10:2375. doi: 10.1038/s41598-020-59382-9 (PMC7012880; doi:10.1038/s41598-020-59382-9)
Supplement: Supplementary file 1 — Supplementary information. [file 41598_2020_59382_MOESM1_ESM.pdf]

Chronic kidney disease attenuates the impact of obesity on quality of life

Sang Heon Suh[1], Hong Sang Choi[1], Chang Seong Kim[1], Eun Hui Bae[1], Seong Kwon Ma[1], Dae Ho Lee[2] and Soo Wan Kim[1]

[1]Department of Internal Medicine, Chonnam National University Medical School, Gwangju, Korea; [2]Department of Internal Medicine, Gachon University College of Medicine, Incheon, Republic of Korea

Correspondence to: Soo Wan Kim

Department of Internal Medicine, Chonnam National University Medical School, 42 Jebongro, Gwangju 61469, Korea

E-mail: skimw@chonnam.ac.kr

Phone: +82-62-220-6271

Fax: +82-62-225-8578

Supplementary information

Table of Contents:

Table S1. Baseline characteristics of the study population categorized by incidence of CKD after propensity score-matching

Table S2. Propensity score-matching analysis of EQ-5D index according to incidence of CKD

Table S3. Mean differences of the EQ-VAS according to obesity parameters in all study subjects and in subgroups stratified by eGFR categories.

Table S4. Mean differences of the EQ-VAS index according to obesity parameters in subgroups stratified by age categories.

Table S5. Baseline characteristics of the study population stratified by BMI quintile categories.

Table S6. Baseline characteristics of the study population stratified by WC quintile categories.

Table S7. Baseline characteristics of the study population stratified by TF quintile categories.

Table S8. Mean differences of the EQ-VAS score according to WC quintiles in all study subjects and in subgroups divided by CKD stages.

Table S9. Mean differences of the EQ-VAS score according to TF quintiles in all study subjects and in subgroups divided by CKD stages.

Table S10. Baseline characteristics of the study population categorized by eGFR with weighted values.

Figure S1. EQ-5D health questionnaire.

Figure S2. Co-linearity examination of the independent variables, hemoglobin and ferritin.

Table S1. Baseline characteristics of the study population categorized by incidence of CKD after propensity score-matching

(mL/min./1.73m2)	CKD (-)	CKD (+)	*p* value
Demographics			
Sample numbers	1640	1640	
Male sex	933 (56.890)	933 (56.890)	
Age	53.954 (17.783)	53.954 (17.783)	
QoL indices			
EQ-5D index	0.922 (0.138)	0.912 (0.151)	0.064
EQ-VAS score	73.209 (18.289)	71.857 (18.526)	0.036
Mobility limitation	352 (21.463)	410 (25.000)	0.0184
Obesity parameters			
BMI (kg/m^2)	23.564 (3.256)	24.230 (3.647)	<0.0001
WC (cm)	81.984 (9.686)	84.094 (11.194)	<0.0001
Truncal fat (kg)	8.749 (3.490)	9.805 (3.855)	<0.0001
TF/WF	0.524 (0.064)	0.539 (0.069)	<0.0001
LF/WF	0.301 (0.056)	0.290 (0.060)	<0.0001

(continued)

Table S1. Baseline characteristics of the study population categorized by incidence of CKD after propensity score-matching (continued)

(mL/min./1.73m2)	CKD (-)	CKD (+)	*p* value
Co-morbidities			
Smoking	823 (50.244)	860 (52.439)	0.2217
Diabetes	149 (9.085)	301 (18.354)	<0.0001
HTN	412 (25.122)	658 (40.122)	<0.0001
Dyslipidemia	151 (9.207)	211 (12.866)	0.001
IHD	44 (2.683)	74 (4.512)	0.0065
Stroke	36 (2.195)	75 (4.573)	0.0002
COPD	15 (1.033)	15 (1.043)	1
Liver cirrhosis	7 (0.427)	6 (0.366)	0.9991
Depression	199 (2.134)	229 (13.963)	0.1328
Laboratory findings			
eGFR (mL/min./1.73m^2)	93.527 (15.797)	82.574 (25.647)	<0.0001
Hgb. (g/dL)	14.191 (1.509)	14.086 (1.790)	0.0707
Ferritin (ng/mL)	98.863 (119.075)	120.182 (219.607)	0.0006
TG (mg/dL)	139.686 (99.959)	156.909 (133.233)	<0.0001
FBS (mg/dL)	98.281 (21.045)	107.319 (36.814)	<0.0001
ALP (IU/L)	232.979 (72.372)	239.272 (79.718)	0.018
PTH (pg/mL)	66.236 (27.680)	77.071 (43.095)	<0.0001
Urine protein≥1+	0 (0.000)	1278 (78.889)	<0.0001

Note: Values for categorical variables are given as number (percentage); values for continuous variables, as mean ± standard deviation. eGFR was calculated using the CKD-EPI creatinine equation. Abbreviations: BMI, body mass index; CAD, coronary artery disease; COPD, chronic obstructive pulmonary disease; eGFR, estimated glomerular filtration rate; LF/WF, ratio of leg fat mass to whole body fat mass; TF/WF, ratio of truncal fat mass to whole body fat mass; WC, waist circumference.

Table S2. Propensity score-matching analysis of EQ-5D index according to incidence of CKD

	Crude		Adjusted	
	Coefficients (95%CIs)	*p* value	Coefficients (95%CIs)	*p* value
BMI				
CKD (-)	0.003 (-0.001,0.006)	0.1305	0.002 (-0.002,0.006)	0.2692
CKD (+)	-0.003 (-0.006,0)	0.0724	-0.003 (-0.006,0.001)	0.1156
WC				
CKD (-)	0.002 (0,0.003)	0.0165	0.001 (-0.001,0.002)	0.2759
CKD (+)	-0.001 (-0.002,0)	0.1284	-0.001 (-0.002,0)	0.0763
TF				
CKD (-)	0.001 (-0.002,0.004)	0.4233	0.004 (0.001,0.007)	0.0121
CKD (+)	-0.006 (-0.009,-0.003)	< 0.001	-0.002 (-0.004,0.001)	0.2316

Note: The analysis model was adjusted for age, sex, comorbidities (diabetes, hypertension, dyslipidemia, coronary artery disease, stroke, chronic obstructive pulmonary disease, and smoking, and laboratory finding (hemoglobin, ferritin, fasting plasma glucose and triglyceride, parathyroid hormone, and alkaline phosphatase). Abbreviations: BMI, body mass index; CI, confidence interval; CKD, chronic kidney disease; LF/WF, ratio of leg fat mass to whole body fat mass; TF, trunctal fat mass; TF/WF, ratio of truncal fat mass to whole body fat mass; WC, waist circumference.

Table S3. Mean differences of the EQ-VAS according to obesity parameters in all study subjects and in subgroups stratified by eGFR categories.

	Crude		Adjusted for model 1		Adjusted for model 2		Adjusted for model 3	
	Coefficients (95%CIs)	*p* value	Coefficients (95%CIs)	*p* value	Coefficients (95%CIs)	*p* value	Coefficients (95%CIs)	*p* value
BMI								
All subjects	-0.053 (-0.147,0.041)	0.2701	-0.015 (-0.109,0.079)	0.753	0.099 (-0.006,0.204)	0.0645	0.213 (0.029,0.398)	0.0238
Group 1	-0.075 (-0.18,0.031)	0.1674	-0.053 (-0.159,0.053)	0.3264	0.061 (-0.06,0.183)	0.3247	0.22 (-0.041,0.48)	0.0993
Group 2	0.185 (-0.019,0.389)	0.0767	0.064 (-0.132,0.26)	0.5223	0.168 (-0.042,0.379)	0.1176	0.229 (-0.033,0.491)	0.0871
Group 3	-0.138 (-0.748,0.471)	0.6567	-0.169 (-0.768,0.43)	0.5802	-0.15 (-0.782,0.482)	0.6422	-0.387 (-1.086,0.312)	0.2793
WC								
All subjects	-0.047 (-0.078,-0.016)	0.0029	-0.032 (-0.066,0.001)	0.0592	-0.002 (-0.038,0.035)	0.9198	0.03 (-0.033,0.094)	0.3484
Group 1	-2.611 (-3.282,-1.939)	< 0.001	-2.469 (-3.207,-1.732)	< 0.001	-0.005 (-0.048,0.038)	0.8156	0.044 (-0.048,0.136)	0.349
Group 2	-6.008 (-7.173,-4.843)	< 0.001	-4.495 (-5.728,-3.262)	< 0.001	0.002 (-0.068,0.072)	0.9565	0.025 (-0.063,0.113)	0.5831
Group 3	-0.041 (-0.264,0.181)	0.715	-0.098 (-0.321,0.125)	0.3882	0.002 (-0.068,0.072)	0.9565	-0.089 (-0.35,0.172)	0.5032
TF								
All subjects	-0.255 (-0.34,-0.169)	< 0.001	-0.09 (-0.174,-0.005)	0.0373	-0.035 (-0.12,0.051)	0.4268	0.122 (-0.052,0.296)	0.1688
Group 1	-0.232 (-0.326,-0.137)	< 0.001	-0.122 (-0.216,-0.029)	0.0106	-0.066 (-0.162,0.03)	0.1771	0.092 (-0.149,0.332)	0.4554
Group 2	-0.199 (-0.383,-0.015)	0.0342	-0.01 (-0.188,0.167)	0.9087	0.053 (-0.127,0.234)	0.5648	0.151 (-0.104,0.406)	0.2457
Group 3	-0.245 (-0.857,0.366)	0.4322	-0.164 (-0.807,0.48)	0.6188	-0.087 (-0.741,0.567)	0.7946	-0.257 (-0.97,0.456)	0.4806

Note: Model 1, adjusted for age and sex. Model 2, Model 1 + adjusted for comorbidities (diabetes, hypertension, dyslipidemia, coronary artery disease, stroke, chronic obstructive pulmonary disease, and smoking). Model 3, model 2 + adjusted for laboratory finding (hemoglobin, ferritin, fasting plasma glucose and triglyceride, parathyroid hormone, alkaline phosphatase, and proteinuria).
Abbreviations: BMI, body mass index; CI, confidence interval; Group 1, ≥ 90 mL/min/1.73 m^2; Group 2, eGFR of 60-89 mL/min/1.73 m^2; Group 3, eGFR < 60 mL/min/1.73 m^2; TF, trunctal fat mass; WC, waist circumference.

Table S4. Mean differences of the EQ-VAS according to obesity parameters in subgroups stratified by age categories.

	Crude		Adjusted for model 1		Adjusted for model 2		Adjusted for model 3	
	Coefficients (95%CIs)	*p* value	Coefficients (95%CIs)	*p* value	Coefficients (95%CIs)	*p* value	Coefficients (95%CIs)	*p* value
BMI								
Age < 60	-0.038 (-0.138,0.063)	0.4615	-0.059 (-0.163,0.045)	0.2654	0.049 (-0.068,0.167)	0.4082	0.347 (0.091,0.602)	0.0081
Age ≥ 60	0.035 (-0.19,0.26)	0.7582	0.055 (-0.165,0.275)	0.6238	0.158 (-0.075,0.391)	0.185	0.09 (-0.157,0.337)	0.4745
WC								
Age < 60	-0.014 (-0.047,0.02)	0.4245	-0.044 (-0.082,-0.007)	0.021	-0.018 (-0.06,0.023)	0.3889	0.069 (-0.025,0.163)	0.151
Age ≥ 60	0.037 (-0.038,0.113)	0.3347	-0.015 (-0.091,0.06)	0.6894	0.019 (-0.061,0.099)	0.6419	-0.006 (-0.092,0.081)	0.8944
TF								
Age < 60	-0.191 (-0.283,-0.1)	< 0.001	-0.131 (-0.223,-0.038)	0.0058	-0.021 (-0.128,0.085)	0.6976	0.207 (-0.04,0.454)	0.1008
Age ≥ 60	-0.211 (-0.411,-0.01)	0.0402	-0.019 (-0.227,0.189)	0.8583	0.097 (-0.122,0.315)	0.3859	0.014 (-0.221,0.249)	0.9057

Note: Model 1, adjusted for age and sex. Model 2, Model 1 + adjusted for comorbidities (diabetes, hypertension, dyslipidemia, coronary artery disease, stroke, chronic obstructive pulmonary disease, and smoking). Model 3, model 2 + adjusted for laboratory finding (hemoglobin, ferritin, fasting plasma glucose and triglyceride, parathyroid hormone, alkaline phosphatase, and proteinuria). Abbreviations: BMI, body mass index; CI, confidence interval; TF, trunctal fat mass; WC, waist circumference.

Table S5. Baseline characteristics of the study population stratified by BMI quintile categories

Varibles	BMI quintile subgroups					*p* value
	1st	2nd	3rd	4th	5th	
BMI ranges (kg/m^2)	>13.7, ≤20.9	>20.9, ≤22.7	>22.7, ≤24.3	>24.3, ≤26.2	>26.2, ≤45.5	
Demographics						
Sample numbers	3395	3398	3394	3397	3395	
Male sex	1205 (35.493)	1440 (42.378)	1619 (47.702)	1733 (51.016)	1652 (48.660)	<0.0001
Age	45.722 ±17.468	49.469 ±16.180	51.239 ±14.901	52.218 ±14.639	51.035 ±14.674	<0.0001
QoL indices						
EQ-5D index	0.945 ± 0.111	0.940 ± 0.120	0.939 ± 0.118	0.928 ± 0.135	0.925 ± 0.132	<0.0001
EQ-VAS score	73.440 ± 17.541)	73.999 ± 17.463	74.469 ± 17.322	74.135 ± 17.742	73.567 ±17.953	0.0991
Mobility limitation	423 ±12.459	480 ± 14.126	533 ±15.704	665 ±19.576	716 ± 21.090	<0.0001
Obesity parameters						
BMI (kg/m^2)	19.372 ± 1.162	21.825 ± 0.524	23.515 ± 0.474	25.242 ± 0.552	28.480 ± 2.179	<0.0001
WC (cm)	69.873 ± 5.546	76.836 ± 5.490	81.526 ± 5.435	85.980 ± 5.677	93.129 ± 7.122	<0.0001
Truncal fat (kg)	5.449 ± 1.822	7.526 ± 2.003	9.012 ± 2.059	10.513 ± 2.127	13.441 ± 3.047	<0.0001
TF/WF	0.456 ± 0.063	0.502 ± 0.061	0.529 ± 0.055	0.548 ± 0.050	0.563 ± 0.046	<0.0001
LF/WF	0.353 ± 0.067	0.320 ± 0.058	0.300 ± 0.051	0.287 ± 0.047	0.280 ± 0.043	<0.0001

(continued)

Table S5. Baseline characteristics of the study population stratified by BMI quintile categories

Varibles	BMI quintile subgroups					*p* value
	1st	2nd	3rd	4th	5th	
BMI ranges (kg/m^2)	>13.7, ≤20.9	>20.9, ≤22.7	>22.7, ≤24.3	>24.3, ≤26.2	>26.2, ≤45.5	
Co-morbidities						
Smoking	2166 (63.913)	2100 (61.965)	1959 (57.788)	1882 (55.434)	1949 (57.510)	<0.0001
Diabetes	130 (3.829)	224 (6.592)	285 (8.397)	333 (9.803)	420 (12.371)	<0.0001
HTN	332 (9.779)	578 (17.010)	767 (22.599)	944 (27.789)	1196 (35.228)	<0.0001
Dyslipidemia	124 (3.652)	239 (7.034)	345 (10.165)	436 (12.835)	527 (15.523)	<0.0001
IHD	37 (1.090)	67 (1.972)	81 (2.387)	90 (2.649)	94 (2.769)	<0.0001
Stroke	36 (1.060)	62 (1.825)	75 (2.210)	73 (2.149)	92 (2.710)	<0.0001
COPD	23 (0.852)	28 (0.974)	16 (0.531)	17 (0.558)	11 (0.369)	0.0258
Liver cirrhosis	9 (0.265)	8 (0.236)	4 (0.118)	6 (0.177)	10 (0.295)	0.5342
Depression	523 (15.405)	483 (14.218)	482 (14.202)	461 (13.571)	507 (14.934)	0.2328
Laboratory findings						
eGFR (mL/min./1.73m^2)	100.842 ± 17.232	96.470 ± 16.560	94.021 ± 15.611	92.441 ± 16.211	93.042 ± 16.637	<0.0001
Hgb. (g/dL)	13.434 ± 1.511	13.770 ± 1.546	14.013 ± 1.557	14.230 ± 1.549	14.346 ± 1.561	<0.0001
Ferritin (ng/mL))	71.791 ± 147.110	78.513 ± 90.036	88.774 ± 149.261	99.823 ± 114.579	103.872 ± 102.278	<0.0001
TG (mg/dL)	96.193 ± 83.939	118.232 ± 93.140	137.059 ± 108.186	157.339 ± 122.078	173.109 ±128.147	<0.0001
FBS (mg/dL)	92.489 ± 23.574	95.387 ± 20.858	97.603 ± 20.121	100.423 ± 21.858	104.592 ± 26.856	<0.0001
ALP (IU/L)	216.958 ± 76.150	222.533 ± 72.847	226.404 ± 68.778	230.611 ± 72.608	235.021 ± 68.888	<0.0001
PTH (pg/mL)	67.023 ± 33.799	64.345 ± 25.880	65.192 ± 27.996	67.318 ± 28.373	71.612 ± 33.166	<0.0001
Urine protein≥1+	251 (7.439)	221 (6.566)	216 (6.425)	251 (7.464)	336 (9.997)	<0.0001

Note: Values for categorical variables are given as number (percentage); values for continuous variables, as mean ± standard deviation. eGFR was calculated using the CKD-EPI creatinine equation. Abbreviations: BMI, body mass index; CAD, coronary artery disease; COPD, chronic obstructive pulmonary disease; eGFR, estimated glomerular filtration rate; LF/WF, ratio of leg fat mass to whole body fat mass; TF/WF, ratio of truncal fat mass to whole body fat mass; WC, waist circumference.

Table S6. Baseline characteristics of the study population stratified by WC quintile categories

Varibles	WC quintile subgroups					*p* value
	1st	2nd	3rd	4th	5th	
WC ranges (cm)	>36.2, ≤72.8	>72.8, ≤78.8	>78.8, ≤84.1	>84.1, ≤89.8	>89.8, ≤166	
Demographics						
Sample numbers	3409	3363	3437	3358	3362	
Male sex	730 (21.414)	1271 (37.794)	1667 (48.502)	1918 (57.117)	2044 (60.797)	<0.001
Age	42.981 ± 16.167	47.738 ± 15.403	51.356 ± 15.010	53.327 ± 14.569	54.379 ± 14.869	<0.001
QoL indices						
EQ-5D index	0.953 ± 0.102	0.942 ± 0.114	0.937 ± 0.115	0.929 ± 0.133	0.914 ± 0.145	<0.001
EQ-VAS score	73.946 ± 16.810	74.527 ± 17.360)	74.821 ± 17.274	73.829 ± 18.139	72.485 ± 18.364	<0.001
Mobility limitation	335 ± 9.827	458 ± 13.619	546 ± 15.886	643 ± 19.148	827 ± 24.598	<0.001
Obesity parameters						
BMI (kg/m^2)	19.973 ± 1.776	22.125 ± 1.728	23.631 ± 1.745	25.047 ± 1.858	27.718 ± 2.754	<0.001
WC (cm)	67.964 ± 3.653	75.895 ± 1.740	81.458 ± 1.524	86.798 ± 1.631	95.455 ± 5.231	<0.001
Truncal fat (kg)	5.842 ± 2.097	7.663 ± 2.395	9.044 ± 2.432	10.401 ± 2.440	13.051 ± 3.195	<0.001
TF/WF	0.446 ± 0.056	0.498 ± 0.054	0.530 ± 0.050	0.554 ± 0.046	0.572 ± 0.043	<0.001
LF/WF	0.366 ± 0.061	0.323 ± 0.052	0.298 ± 0.047	0.281 ± 0.043	0.271 ± 0.039	<0.001

(continued)

Table S6. Baseline characteristics of the study population stratified by WC quintile categories (continued)

Varibles	WC quintile subgroups					*p* value
	1st	2nd	3rd	4th	5th	
WC ranges (cm)	>36.2, ≤72.8	>72.8, ≤78.8	>78.8, ≤84.1	>84.1, ≤89.8	>89.8, ≤166	
Co-morbidities						
Smoking	2578 (75.757)	2203 (65.605)	1969 (57.372)	1691 (50.447)	1580 (47.066)	<0.001
Diabetes	85 (2.493)	142 (4.222)	264 (7.681)	372 (11.078)	526 (15.645)	<0.001
HTN	245 (7.187)	505 (15.016)	722 (21.007)	1008 (30.018)	1325 (39.411)	<0.001
Dyslipidemia	116 (3.403)	235 (6.988)	325 (9.456)	461 (13.728)	531 (15.794)	<0.001
IHD	29 (0.851)	43 (1.279)	72 (2.095)	106 (3.157)	118 (3.510)	<0.001
Stroke	22 (0.645)	44 (1.308)	73 (2.124)	91 (2.710)	106 (3.153)	<0.001
COPD	15 (0.570)	21 (0.743)	21 (0.687)	21 (0.696)	17 (0.558)	0.877
Liver cirrhosis	4 (0.117)	7 (0.208)	10 (0.291)	6 (0.179)	10 (0.297)	0.456
Depression	538 (15.786)	507 (15.076)	481 (13.995)	451 (13.431)	468 (13.920)	0.038
Laboratory findings						
eGFR (mL/min./1.73m^2)	102.613 ± 16.197	98.072 ± 15.645	93.983 ± 15.542	92.005 ± 16.042	90.053 ± 17.130	<0.001
Hgb. (g/dL)	13.208 ± 1.409	13.672 ± 1.546	14.050 ± 1.541	14.319 ± 1.518	14.558 ± 1.508	<0.001
Ferritin (ng/mL))	53.575 ± 62.593	74.680 ± 32.617	90.631 ± 107.251	105.439 ±163.243	119.206 ±120.146	<0.001
TG (mg/dL)	85.757 ± 53.494	115.785 ± 90.354	139.640 ±110.244	159.735 ±123.894	181.847 ±135.929	<0.001
FBS (mg/dL)	90.204 ± 15.639	94.511 ± 22.603	97.931 ± 21.627	101.227 ± 22.791	106.773 ± 28.059	<0.001
ALP (IU/L)	206.531 ± 71.830	219.497 ± 69.328	230.653 ± 71.199)	234.770 ± 70.101	240.385 ± 72.268	<0.001
PTH (pg/mL)	66.112 ± 33.075	65.040 ± 26.417	65.921 ± 28.203	66.975 ± 30.920	70.284 ± 30.484	<0.001
Urine protein≥1+	242 (7.158)	195 (5.844)	215 (6.303)	231 (6.945)	387 (11.653)	<0.001

Note: Values for categorical variables are given as number (percentage); values for continuous variables, as mean ± standard deviation. eGFR was calculated using the CKD-EPI creatinine equation. Abbreviations: BMI, body mass index; CAD, coronary artery disease; COPD, chronic obstructive pulmonary disease; eGFR, estimated glomerular filtration rate; LF/WF, ratio of leg fat mass to whole body fat mass; TF/WF, ratio of truncal fat mass to whole body fat mass; WC, waist circumference.

Table S7. Baseline characteristics of the study population stratified by TF quintile categories

Varibles	WC quintile subgroups					*p* value
	1st	2nd	3rd	4th	5th	
TF ranges (kg)	>1.32, ≤6.09	>6.09, ≤8.11	>8.11, ≤9.86	>9.86, ≤12.0	>12.0, ≤29.2	
Demographics						
Sample numbers	3400	3400	3400	3400	3400	
Male sex	1967 (57.853)	1649 (48.500)	1554 (45.706)	1378 (40.529)	1111 (32.676)	<0.0001
Age	46.178 ± 17.528	48.188 ± 15.801	50.758 ± 14.998	52.154 ± 14.570	52.462 ± 14.890	<0.0001
QoL indices						
EQ-5D index	0.946 ± 0.113	0.944 ± 0.115	0.938 ± 0.123	0.931 ± 0.124	0.917 ± 0.139	<0.0001
EQ-VAS score	74.243 ± 17.292	74.641 ± 17.257	74.426 ± 17.266	73.918 ± 17.485	72.344 ± 18.669	<0.0001
Mobility limitation	428 ± 12.588)	452 ± 13.294	528 ± 15.529	626 ± 18.412	791 ± 23.265	<0.0001
Obesity parameters						
BMI (kg/m^2)	20.191 ± 1.958	22.173 ± 1.942	23.502 ± 1.892	24.896 ± 1.895	27.675 ± 2.785	<0.0001
WC (cm)	71.689 ± 6.634	77.347 ± 7.267	81.275 ± 6.882	85.061 ± 6.598	91.989 ± 7.877	<0.0001
Truncal fat (kg)	4.596 ± 1.070	7.140 ± 0.585	8.985 ± 0.506	10.854 ± 0.602	14.371 ± 2.226	<0.0001
TF/WF	0.451 ± 0.060	0.498 ± 0.057	0.531 ± 0.052	0.549 ± 0.047	0.569 ± 0.044	<0.0001
LF/WF	0.347 ± 0.069	0.324 ± 0.060	0.301 ± 0.052	0.289 ± 0.046	0.278 ± 0.042	<0.0001

(continued)

Table S7. Baseline characteristics of the study population stratified by TF quintile categories (continued)

Varibles	TF quintile subgroups					p value
	1st	2nd	3rd	4th	5th	
TF ranges (kg)	>1.32, ≤6.09	>6.09, ≤8.11	>8.11, ≤9.86	>9.86, ≤12.0	>12.0, ≤29.2	
Co-morbidities						
Smoking	1708 (50.339)	1910 (56.226)	1988 (58.539)	2133 (62.828)	2330 (68.691)	<0.0001
Diabetes	133 (3.912)	195 (5.735)	299 (8.794)	316 (9.294)	451 (13.265)	<0.0001
HTN	345 (10.147)	512 (15.059)	796 (23.412)	910 (26.765)	1265 (37.206)	<0.0001
Dyslipidemia	96 (2.824)	207 (6.088)	335 (9.853)	434 (12.765)	603 (17.735)	<0.0001
IHD	38 (1.118)	45 (1.324)	88 (2.588)	95 (2.794)	103 (3.029)	<0.0001
Stroke	38 (1.118)	53 (1.559)	79 (2.324)	77 (2.265)	92 (2.706)	<0.0001
COPD	24 (0.840)	19 (0.668)	17 (0.577)	23 (0.762)	12 (0.404)	0.2648
Liver cirrhosis	9 (0.265)	9 (0.265)	5 (0.147)	5 (0.147)	9 (0.265)	<0.0001
Depression	394 (11.588)	458 (13.471)	494 (14.534)	529 (15.559)	583 (17.147)	<0.0001
Laboratory findings						
eGFR (mL/min./1.73m^2)	99.063 ± 16.328	96.980 ± 16.439	94.701 ± 16.444	93.081 ± 16.584	92.953 ± 17.092	<0.0001
Hgb. (g/dL)	13.907 ± 1.627	13.928 ± 1.605	13.962 ± 1.592	13.992 ± 1.569	14.007 ± 1.499	0.048
Ferritin (ng/mL))	83.450 ± 145.723	86.213 ± 110.012	89.294 ± 159.613)	92.189 ± 96.379	91.756 ± 90.464	0.0085
TG (mg/dL)	96.467 ± 88.168	124.329 ± 113.895	142.907 ± 117.835	152.945 ± 103.903	165.345 ± 118.871	<0.0001
FBS (mg/dL)	92.555 ± 22.187	95.231 ± 20.516	98.250 ± 22.524	100.344 ± 22.727	104.135 ± 25.734	<0.0001
ALP (IU/L)	221.289 ± 75.969	219.637 ± 71.636	224.127 ± 71.062	229.586 ± 69.750	237.048 ± 71.262	<0.0001
PTH (pg/mL)	64.867 ± 32.098	65.632 ± 27.317	63.973 ± 26.371	68.310 ± 32.739	71.534 ± 29.716	<0.0001
Urine protein≥1+	250 (7.405)	238 (7.056)	225 (6.651)	229 (6.813)	336 (10.018)	<0.0001

Note: Values for categorical variables are given as number (percentage); values for continuous variables, as mean ± standard deviation. eGFR was calculated using the CKD-EPI creatinine equation. Abbreviations: BMI, body mass index; CAD, coronary artery disease; COPD, chronic obstructive pulmonary disease; eGFR, estimated glomerular filtration rate; LF/WF, ratio of leg fat mass to whole body fat mass; TF/WF, ratio of truncal fat mass to whole body fat mass; WC, waist circumference.

Table S8. Mean differences of the EQ-VAS score according to WC quintiles in all study subjects and in subgroups divided by CKD stages

	Crude		Adjusted for model 3	
	Coefficients (95%CIs)	*p* value	Coefficients (95%CIs)	*p* value
All subjects				
WC, 1st quintile	Reference		Reference	
WC, 2nd quintile	1.318 (0.413,2.222)	0.0045	0.044 (-2.016,2.104)	0.967
WC, 3rd quintile	1.146 (0.191,2.102)	0.0191	2.294 (0.386,4.202)	0.0188
WC, 4th quintile	0.162 (-0.777,1.101)	0.7352	1.724 (-0.229,3.677)	0.0841
WC, 5th quintile	-0.888 (-1.802,0.026)	0.0574	0.952 (-1.136,3.04)	0.3718
Group 1				
WC, 1st quintile	Reference		Reference	
WC, 2nd quintile	1.572 (0.623,2.521)	0.0012	0.855 (-1.797,3.508)	0.5276
WC, 3rd quintile	1.528 (0.485,2.57)	0.0042	3.709 (1.247,6.172)	0.0033
WC, 4th quintile	0.363 (-0.707,1.433)	0.5064	2.685 (0.147,5.224)	0.0387
WC, 5th quintile	-0.626 (-1.688,0.436)	0.2483	1.315 (-1.45,4.08)	0.3516
Group 2				
WC, 1st quintile	Reference		Reference	
WC, 2nd quintile	1.22 (-0.918,3.359)	0.2638	-0.963 (-3.826,1.899)	0.5097
WC, 3rd quintile	1.764 (-0.326,3.855)	0.0987	0.756 (-1.865,3.376)	0.5721
WC, 4th quintile	1.506 (-0.537,3.548)	0.1492	0.605 (-2.029,3.239)	0.6526
WC, 5th quintile	0.686 (-1.251,2.623)	0.4881	0.39 (-2.397,3.176)	0.784
Group 3				
WC, 1st quintile	Reference		Reference	
WC, 2nd quintile	-0.776 (-11.106,9.554)	0.883	2.648 (-9.802,15.098)	0.6772
WC, 3rd quintile	-0.77 (-9.57,8.03)	0.864	0.673 (-9.283,10.629)	0.8947
WC, 4th quintile	0.627 (-7.671,8.924)	0.8825	1.213 (-9.488,11.915)	0.8244
WC, 5th quintile	-0.751 (-8.471,6.968)	0.8489	0.152 (-9.911,10.215)	0.9765

Note: Model 3, adjusted for age, sex (Model 1), comorbidities (diabetes, hypertension, dyslipidemia, coronary artery disease, stroke, chronic obstructive pulmonary disease, and smoking, Model 2), and laboratory finding (hemoglobin, ferritin, fasting plasma glucose and triglyceride, parathyroid hormone, alkaline phosphatase, proteinuria, and eGFR). Abbreviations: CI, confidence interval; Group 1, ≥ 90 mL/min/1.73 m^2; Group 2, eGFR of 60-89 mL/min/1.73 m^2; Group 3, eGFR < 60 mL/min/1.73 m^2; WC, waist circumference.

Table S9. Mean differences of the EQ-VAS score according to TF quintiles in all study subjects and in subgroups divided by CKD stages

	Crude		Adjusted for model 3	
	Coefficients (95%CIs)	*p* value	Coefficients (95%CIs)	*p* value
All subjects				
TF, 1st quintile	Reference		Reference	
TF, 2nd quintile	0.072 (-0.758,0.902)	0.8646	1.724 (0.093,3.354)	0.0388
TF, 3rd quintile	0.033 (-0.833,0.899)	0.9405	2.661 (0.903,4.419)	0.0032
TF, 4th quintile	-0.927 (-1.834,-0.019)	0.0458	3.401 (1.663,5.139)	< 0.001
TF, 5th quintile	-2.295 (-3.269,-1.321)	< 0.001	1.659 (-0.271,3.59)	0.0927
Group 1				
TF, 1st quintile	Reference		Reference	
TF, 2nd quintile	0.06 (-0.819,0.939)	0.8941	1.885 (-0.34,4.109)	0.0975
TF, 3rd quintile	-0.388 (-1.354,0.577)	0.4306	2.294 (-0.199,4.787)	0.0719
TF, 4th quintile	-0.845 (-1.859,0.168)	0.1028	4.176 (1.817,6.535)	< 0.001
TF, 5th quintile	-1.967 (-3.038,-0.896)	< 0.001	1.475 (-1.125,4.076)	0.2667
Group 2				
TF, 1st quintile	Reference		Reference	
TF, 2nd quintile	1.692 (-0.7,4.084)	0.1662	1.504 (-0.914,3.921)	0.2234
TF, 3rd quintile	2.116 (-0.372,4.605)	0.0962	2.609 (0.119,5.098)	0.0405
TF, 4th quintile	1.403 (-1.146,3.952)	0.2812	2.439 (-0.178,5.056)	0.0684
TF, 5th quintile	-0.787 (-3.382,1.807)	0.5522	1.765 (-1.063,4.594)	0.2218
Group 3				
TF, 1st quintile	Reference		Reference	
TF, 2nd quintile	1.139 (-7.174,9.452)	0.7885	4.442 (-3.741,12.625)	0.2887
TF, 3rd quintile	7.824 (0.456,15.191)	0.0385	5.954 (-2.113,14.02)	0.1495
TF, 4th quintile	2.573 (-5.503,10.65)	0.5329	3.535 (-4.939,12.008)	0.4145
TF, 5th quintile	-0.307 (-7.269,6.654)	0.9311	0.711 (-7.118,8.541)	0.8588

Note: Model 3, adjusted for age, sex (Model 1), comorbidities (diabetes, hypertension, dyslipidemia, coronary artery disease, stroke, chronic obstructive pulmonary disease, and smoking, Model 2), and laboratory finding (hemoglobin, ferritin, fasting plasma glucose and triglyceride, parathyroid hormone, alkaline phosphatase, proteinuria and eGFR). Abbreviations: CI, confidence interval; Group 1, ≥ 90 mL/min/1.73 m^2; Group 2, eGFR of 60-89 mL/min/1.73 m^2; Group 3, eGFR < 60 mL/min/1.73 m^2; TF, truncal fat mass.

Table S10. Baseline characteristics of the study population categorized by eGFR with weighted values

(mL/min./1.73m2)	Group 1 (eGFR≥90)	Group 2 (60≤eGFR<90)	Group 3 (eGFR<60)	*p* value
Demographics				
Sample numbers	22905746.48	8592188.058	623903.249	
Male sex	11563429.418 (50.483)	4953175.645 (57.647)	334589.239 (53.628)	<0.001
Age	40.488 (12.729)	57.041 (13.890)	69.631 (10.346)	<0.001
QoL indices				
EQ-5D index	0.961 ± 0.086	0.923 ± 0.138	0.842 ± 0.195	<0.001
EQ-VAS score	75.589 ± 15.438	73.351 ± 18.759	67.626 ± 20.971	<0.001
Mobility limitation	1.081 ± 0.278	1.220 ± 0.431	1.515 ± 0.560	<0.001
Obesity parameters				
BMI (kg/m^2)	23.495 ± 3.446	24.171 ± 3.107	24.543 ± 3.232	<0.001
WC (cm)	80.256 ± 10.012	83.774 ± 9.244	86.498 ± 9.358	<0.001
Truncal fat (kg)	8.884 ± 3.654	9.427 ± 3.405	10.309 ± 3.380	<0.001
TF/WF	0.506 ± 0.067	0.541 ± 0.059	0.564 ± 0.056	<0.001
LF/WF	0.320 ± 0.061	0.287 ± 0.050	0.269 ± 0.046	<0.001

(continued)

Table S10. Baseline characteristics of the study population categorized by eGFR with weighted values

(mL/min./1.73m2)	Group 1 (eGFR≥90)	Group 2 (60≤eGFR<90)	Group 3 (eGFR<60)	*p* value
Co-morbidities				
Smoking	10096871.380 (44.129)	4312427.977 (50.321)	326052.023 (52.260)	<0.001
Diabetes	938469.219 (4.097)	917148.600 (10.674)	200577.521 (32.149)	<0.001
HTN	2611481.229 (11.401)	2632675.218 (30.640)	436884.619 (70.024)	<0.001
Dyslipidemia	1476369.077 (6.445)	1063505.998 (12.378)	141577.211 (22.692)	<0.001
IHD	184812.646 (0.807)	252162.957 (2.935)	61782.435 (9.903)	<0.001
Stroke	131457.793 (0.574)	249965.495 (2.909)	58719.200 (9.412)	<0.001
COPD	80969.777 (0.467)	60523.292 (0.751)	11013.485 (1.855)	0.009
Liver cirrhosis	38789.621 (0.169)	27041.225 (0.315)	1372.006 (0.220)	0.173
Depression	2851465.975 (12.449)	1228026.403 (14.295)	96778.068 (15.512)	0.009
Laboratory findings				
eGFR (mL/min./1.73m^2)	106.150 ± 10.160	80.455 ± 7.458	48.539 ± 11.452	<0.001
Hgb. (g/dL)	14.166 ± 1.609	14.322 ± 1.539	13.104 ± 1.864	<0.001
Ferritin (ng/mL))	87.941 ± 108.990	104.138 ±158.832	116.658 ±131.262	<0.001
TG (mg/dL)	131.379 ± 114.643	150.272 ±116.713	171.141 ±104.034	<0.001
FBS (mg/dL)	95.282 ± 20.801	100.717 ± 23.856	109.324 ± 34.372	<0.001
ALP (IU/L)	217.822 ± 67.379	235.735 ± 71.094	246.632 ± 77.763	<0.001
PTH (pg/mL)	64.812 ± 26.559	66.948 ± 27.122	86.627 ± 53.503	<0.001
Urine protein≥1+	1784444.712 (7.790)	726402.405 (8.685)	147974.112 (24.667)	<0.001

Note: Values for categorical variables are given as number (percentage); values for continuous variables, as mean ± standard deviation. eGFR was calculated using the CKD-EPI creatinine equation. Abbreviations: BMI, body mass index; CAD, coronary artery disease; COPD, chronic obstructive pulmonary disease; eGFR, estimated glomerular filtration rate; LF/WF, ratio of leg fat mass to whole body fat mass; TF/WF, ratio of truncal fat mass to whole body fat mass; WC, waist circumference.

By placing a tick in one box in each group below, please indicate which statements best describe your own health state today.

Mobility

I have no problems in walking about ☐
I have some problems in walking about ☐
I am confined to bed ☐

Self-care

I have no problems with self-care ☐
I have some problems washing or dressing myself ☐
I am unable to wash or dress myself ☐

Usual activities *(e.g. work, study, housework, family or leisure activities)*

I have no problems with performing my usual activities ☐
I have some problems with performing my usual activities ☐
I am unable to perform my usual activities ☐

Pain/discomfort

I have no pain or discomfort ☐
I have moderate pain or discomfort ☐
I have extreme pain or discomfort ☐

Anxiety/depression

I am not anxious or depressed ☐
I am moderately anxious or depressed ☐
I am extremely anxious or depressed ☐

Figure S1. EQ-5D health questionnaire.

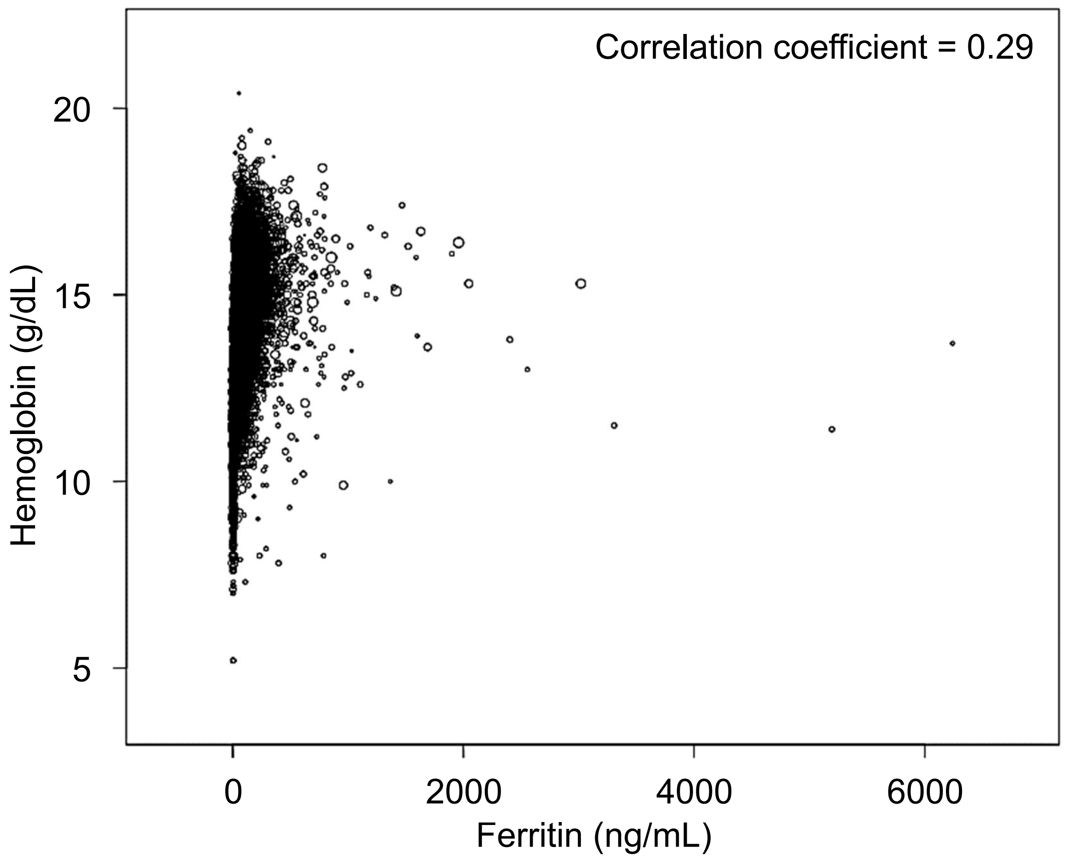

Figure S2. Co-linearity examination of the independent variables, hemoglobin and ferritin.
